# A Cell-Free Approach Based on Phospholipid Characterization for Determination of the Cell Specific Unbound Drug Fraction (f_u,cell_)

**DOI:** 10.1007/s11095-019-2717-1

**Published:** 2019-11-07

**Authors:** Andrea Treyer, Sandra Walday, Hinnerk Boriss, Pär Matsson, Per Artursson

**Affiliations:** 1 Department of Pharmacy, Uppsala University, Box 580, SE-751 23 Uppsala, Sweden; 2 HBO consult GmbH, 04155 Leipzig, Germany; 3 Science for Life Laboratory Drug Discovery and Development platform (SciLifelab DDD-P), Uppsala, Sweden; 4 Uppsala University Drug Optimization and Pharmaceutical Profiling Platform (UDOPP), Uppsala University, Uppsala, Sweden

**Keywords:** cell-free assays, intracellular bioavailability, phospholipid membranes, unbound drug fraction

## Abstract

**Purpose:**

The intracellular fraction of unbound compound (f_u,cell_) is an important parameter for accurate prediction of drug binding to intracellular targets. f_u,cell_ is the result of a passive distribution process of drug molecules partitioning into cellular structures. Initial observations in our laboratory showed an up to 10-fold difference in the f_u,cell_ of a given drug for different cell types. We hypothesized that these differences could be explained by the phospholipid (PL) composition of the cells, since the PL cell membrane is the major sink of unspecific drug binding. Therefore, we determined the f_u,cell_ of 19 drugs in cell types of different origin.

**Method:**

The cells were characterized for their total PL content and we used mass spectrometric PL profiling to delineate the impact of each of the four major cellular PL subspecies: phosphatidylcholine (PC), phosphatidylethanolamine (PE), phosphatidylserine (PS) and phosphatidylinositol (PI). The cell-based experiments were compared to cell-free experiments that used beads covered by PL bilayers consisting of the most abundant PL subspecies.

**Results:**

PC was found to give the largest contribution to the drug binding. Improved correlations between the cell-based and cell-free assays were obtained when affinities to all four major PL subspecies were considered. Together, our data indicate that f_u,cell_ is influenced by PL composition of cells.

**Conclusion:**

We conclude that cellular PL composition varies between cell types and that cell-specific mixtures of PLs can replace cellular assays for determination of f_u,cell_ as a rapid, small-scale assay covering a broad dynamic range.

**Graphical Abstract:**
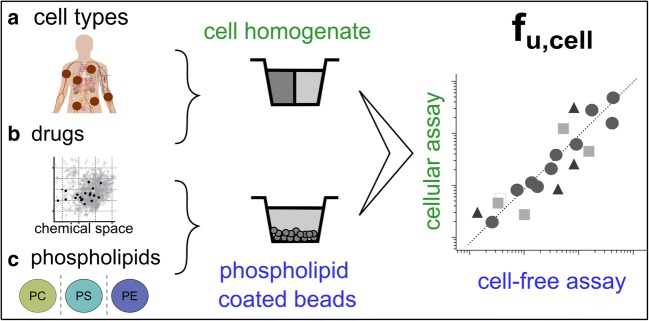
.

**Electronic supplementary material:**

The online version of this article (10.1007/s11095-019-2717-1) contains supplementary material, which is available to authorized users.

## Introduction

Determination of intracellular unbound drug concentrations has gained importance over the last decade (1,2). Several techniques for determination of the intracellular unbound drug accumulation ratio at steady-state (Kp_uu_) or intracellular bioavailability (F_ic_) have been published recently and have been summarized by the international transporter consortium (3). Many of the techniques determine the total accumulation ratio at equilibrium (Kp) and the fraction unbound to cells (f_u,cell_), which are combined to calculate Kp_uu_ or F_ic_. Kp is generally determined by performing drug uptake experiments in suspended or plated cells. The most prominent techniques for determining f_u,cell_ are the binding/homogenization method, temperature method, or predictions from logD_7.4_ (1,4).

Mechanisms such as active and passive transport processes across cell membranes, as well as partitioning into substructures of the cell (e.g., lysosomal trapping) are relatively well understood. However, the subcellular structures that determine f_u,cell_ have not been investigated in detail. The current study investigates previously observed differences of f_u,cell_ between different cell types. More specifically, we observed a lower f_u,cell_ in human hepatocytes than in HEK293 cells, consistent with an approximately 5-fold greater binding capacity of the former, but we did not identify the factors behind this difference (5). More recently, we showed that induction of the phospholipid (PL) content in 3T3-L1 cells results in a corresponding increase in drug binding. In contrast, no increase in f_u,cell_ was observed after a 5-fold enhancement of the cellular content of neutral lipids (6). Global quantitative proteomics analysis allowed us to investigate the relative importance of intracellular drug binding proteins such as fatty acid binding proteins. These comparisons show that PLs are the major sink for unspecific drug binding, and dominate over protein binding (6). PL membranes are complex structures, and little is known about the binding mechanisms of drugs towards the different PL subtypes.

In the present study, we therefore compared the f_u,cell_ of 19 chemically diverse drugs in cell types derived from different tissues (lung, intestine, liver, kidney, blood, and bone marrow). We next determined the PL composition in each of the cell types by mass spectrometric PL profiling. We then constructed beads covered with PL membranes, consisting of single PL subspecies as well as a PL-mixture, to represent the membrane of a prototypical cell. This allowed us to determine the contribution of each PL subspecies to cellular drug binding separately. The f_u,cell_ values were then compared to binding affinities measured with the bead-immobilized membranes of defined PL composition. We speculated that a PL-based cell-free assay that could predict f_u,cell_ and still take the cell specific lipid composition into account would provide an advantage over the current cell-based methodology. Moreover, membrane dialysis can be a limiting step for very large or very lipophilic compounds that cannot pass the pores in the dialysis membrane. A better understanding of the influence of different cellular constituents on f_u,cell_ would enable the development of alternative methods that overcome these challenges.

## Materials and Methods

### Chemicals

Compounds were purchased from Fluka (caffeine, phenazopyridine), Baxter (esmolol), Therapeutic Research Center (fluconazole) or Sigma Aldrich (remaining compounds) at their highest available purity (>95%). The compounds were dissolved at a concentration of 10 mM in DMSO or at their highest possible solubility if lower. DMSO stocks were kept at −20°C.

### Compound Selection

Compounds were selected to represent the common chemical space of small-molecule drugs, based on a principal component analysis of 334 ADME-related molecular properties using ADMET predictor (Simulations Plus, version 7.2), as described elsewhere (6). The final compound set comprised compounds representing all charge classes and with molecular weight (MW), polar surface area (PSA) and lipophilicity (logD_7.4_) ranging from 194 to 629, 28 to 146 Å and − 0.7 to 5.0, respectively (Table S1).

### Cell Culture

Cells were grown in T75 culture flasks at 37°C and 5% CO_2_ atmosphere (10% for Caco-2 cells) and passaged and/or harvested at 80-100% confluency. Suspended cells were maintained at a cell density between 1 × 10^5^ and 1 × 10^6^ viable cells/ml. HEK293 and A549 cells were grown in Dulbecco’s Modified Eagle’s Medium (DMEM) supplemented with 10% fetal bovine serum (FBS), 1% penicillin-streptomycin (PEST) and 2 mM L-glutamine. MDCK cells were grown in DMEM supplemented with 10% FBS, 1% PEST and 1% Glutamax. LLC-PK1 cells were grown in medium 199 supplemented with 10% FBS, 1% PEST and 2 mM L-glutamine. K562 and HL60 cells were grown in Roswell Park Memorial Institute (RPMI) 1640 medium supplemented with 10% FBS, 1% PEST and 2 mM L-glutamine and Caco-2 in DMEM supplemented with 10% FBS and 1% nonessential amino acids. After trypsinisation, cells were pelleted at 10-30 million cells per flask and stored at −20°C until further processing. Primary human hepatocytes (HH) were frozen directly after isolation from human liver tissue using a two-step collagenase procedure as described elsewhere (7,8). Ethical approval was granted by the Uppsala Regional Ethics Committee (ethical approvals no. 2009/028 and 2011/037).

### Determination of f_u,cell_

f_u,cell_ was measured in cassette-mode as previously described with minor modifications (9). Briefly, 10 million cells/mL were suspended in HBSS buffered with 10 mM HEPES and homogenized on ice by sonication (VCX 750 Sonicator, 3 mm probe, 20% intensity, 10 s). Up to 8 compounds were combined randomly and spiked into the homogenate for a final concentration of 0.5 μM. Equilibrium dialysis was performed in a Rapid Equilibirum device (Thermo Fisher Scientific) against blank HBSS buffered with 10 mM HEPES, for 4 h at 37°C on an orbital shaker at 900 rpm. Stability controls were kept at 4° and 37°C for the duration of the experiment. The concentration in both dialysis chambers was quantified by extracting the compound with acetonitrile/water (60/40) containing internal standard. Matrixes were matched with blank buffer or cell homogenate, respectively. LC-MS/MS parameters are available in Table S9. All experiments were carried out in triplicates and at least at two independent occasions.

The unbound fraction in the cell homogenate (f_u,hom_) was determined according to Eq. (1):

1 $$ {f}_{u,\mathit{\hom}}=\frac{C_{buffer}}{C_{hom}} $$

and the fraction of unbound compound in the cell (f_u,cell_) was calculated by correcting for homogenate dilution according to Eq. (2):

2 $$ {f}_{u, cell}=\frac{1}{D_P\bullet \left(1/{f}_{u,\mathit{\hom}}-1\right)+1} $$

where the dilution constant D_P_ was calculated using Eq. (3), assuming the V_cell_ to be equal to 6.5 μL/mg protein (6,10). Protein content in the cell homogenates was determined at each experiment using the BCA Protein Assay Reagent Kit (Thermo Fisher Scientific Inc.).

3 $$ {D}_P=1/{V}_{cell} $$

### Determination of f _u,PL_

f _u,PL_ was derived from membrane affinity measurements to beads covered with PLs as previously described (6). PL-covered silica beads with PC (Sovicell, Transil Absorption Kit, No. TMP-0100-2096), PE (no. TMP-0130-2096), PS (no. TMP-0140-2096) or PE/PS/PI/PC (21.6/12/14.3/52.1 mol%) (no. TMP-0150-2096) were used to determine membrane affinity, defined as (4)

4 $$ membrane\ affinity=\frac{C_{membrane}}{C_{buffer}} $$

C_buffer_ was quantified by LC-MS/MS in samples of the supernatant that was obtained after 15 min incubation time on an orbital shaker at 1000 rpm and subsequent separation from the beads by centrifugation at 750 x *g*. C_membrane_ was calculated taking into account the volume of the lipid membrane (90 μl) using the provided software from the Absorption Kit (TMP-0100-2096) and the mass balance equation:

5 $$ n={c}_b\bullet {V}_b+{c}_m\bullet {V}_m $$

where n: total amount of drug, c: concentration, V: volume, b: buffer, m: membrane. Unspecific binding was evaluated by incubations into wells without added lipids.

Finally, f_u,PL_ was derived as follows:

6 $$ {f}_{u, PL}=\frac{C_{buffer}}{C_{buffer}+{C}_{membrane}} $$

### Prediction of f_u,hom_

f_u,hom,pred_ was calculated by scaling f _u,PL_ from the pure PL system to the homogenates, applying an optimized dilution factor (D_L_) determined by minimizing the sum of the squared prediction errors (Microsoft Excel, Solver add-in, version 16.0):

7 $$ {f}_{u,\mathit{\hom}, pred}=\frac{1}{D_L\bullet \left(1/{f}_{u, PL}-1\right)+1} $$

### Phospholipid Content in Cell Homogenates

The PL content of cell homogenates was quantified using the enzymatic-colorimetric WAKO LabAssay Phospholipid Choline Oxidase/DAOS method (Nordic Biolabs) according to the manufacturer’s instructions. Briefly, 2 μl of the cell homogenate and the provided standards were deposited in a 96-well black, clear-bottom plate and 300 μl of colour reagent was added prior to incubation at 37°C for 5 min. Absorbance was measured for multiple reads per well at 600 nm in a plate reader.

### ESI-MS/MS Based Quantification of Phospholipid Subspecies

Proportional content of the PL subspecies was determined using a shotgun lipidomic approach. The lipids were extracted from the cellular homogenates by a liquid-liquid extraction. This extraction method, using methyl-*tert*-butyl ether (MTBE) as organic solvent, gives recoveries of approximately 90% for several PL subspecies (11). Cell pellets were suspended at 50 million cells per ml and homogenized by sonication (VCX 750 Sonicator, 3 mm probe, 20% intensity, 10 s). The sample (500 μL) was transferred to a glass vial to which 1800 μL of high-grade methanol was added. After vortexing, 6 ml of MTBE was added, and the samples were shaken on an orbital shaker at 600 rpm for 1 h. Phase separation was induced by adding 1250 μL of purified water. After 5 min, the samples were centrifuged for 10 min at 1000x *g* and the upper organic phase was separated using a glass pipette. The sample was re-extracted by adding artificial organic phase (MTBE:methanol:water at 4:1.2:1, *v/v/v*) to the water phase. After pooling the organic phases from both steps together, the solvent was evaporated on a vacuum centrifuge (EZ-2 MK2 Plus centrifugal evaporator, Genevac Ltd., Ipswich, England). Samples were stored under inert atmosphere at −80°C if not processed immediately. For MS analysis, the samples were dissolved in 200 μL analysis buffer consisting of isopropanol, methanol and water (5:1:4, *v/v/v*) containing 0.2% (v/v) formic acid and 0.028% (*w*/*v*) ammonium acetate (12).

For mass-spectrometry based PL profiling, mixtures were further diluted 1:1000 in analysis buffer and infused at a flow rate of 0.1 ml/min into the ion source of a Sciex QTRAP 6500 mass spectrometer using a glass syringe. Spectra of specific fragments of each PL subspecies were acquired simultaneously, using precursor ion scan (184 Da m/z for PC and SM) or neutral loss scan (141 Da m/z for PE, 185 Da m/z for PS, 98 Da m/z for PA, 277 Da m/z for PI and 172 Da m/z for PG) in positive mode (13,14). Ion intensities were exported from Analyst 1.6.2 software (AB Sciex, Framingham, MA, USA), then summed up and normalized against intensities from a standard of known concentration for each subspecies of PLs (the mixture came from the Differential Ion Mobility System Suitability LIPIDOMIX kit, no. 330708, Avanti, Alabama, USA).

### Calculation of Molecular Properties

Chemical structures of the study compounds were accessed from DrugBank (15) or PubChem (http://pubchem.ncbi.nlm.nih.gov) in SMILES format. Three-dimensional structures were generated using Corina (Molecular Networks, version 4.1) and were used as input for molecular property calculations using the ADMET Predictor (Simulations Plus, version 7.2).

#### Statistical Analysis

All statistical analyses were performed in Graph-Pad Prism (version 7.04). R^2^ and root-mean square error (RMSE) were calculated using the linear regression function. For correlations between lipid content and f_u,cell_, the slope of the linear regression was considered significantly non-zero at a *p* value <0.05. f_u,cell_ experiments were carried out in triplicates and were performed on at least two independent occasions.

## Results

### Comparison of f_u,cell_ between Cell Types

**Fig. 1:**
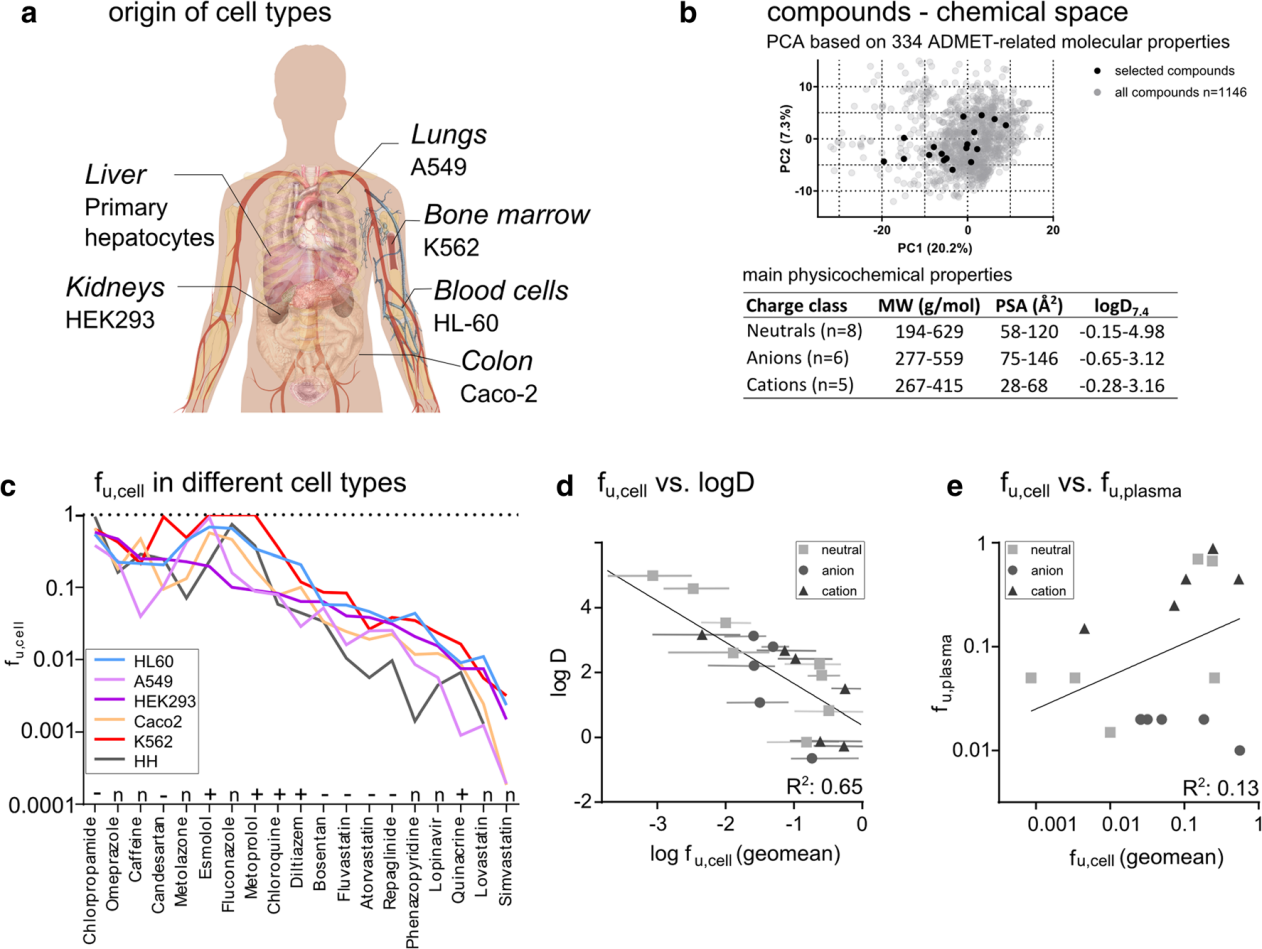
(**a**) Origin of the cell types tested. (**b**) Selection and properties of compounds tested (for compounds and compound properties see Table S1 and Fig. S2). (**c**) Overview of f_u,cell_ across the human cell types, sorted by decreasing f_u,cell_ in HEK293 cells. For simplicity in the presentation, only geometric mean values without standard deviations are shown. Full information is available in Table S4. (**d**) Geometric mean of f_u,cell_ of each compound across all cell types plotted against logD. Lines indicate maximum and minimum values of f_u,cell_ (**e**) Geometric mean of f_u,cell_ of each compound across all cell types plotted against f_u,plasma_ derived from DrugBank (15).

The fraction of unbound drug in cells (f_u,cell_) was determined in six cell types originating from different human tissues (Fig. 1a) using equilibrium dialysis of drug added to cell homogenates, as described previously (5,16). In addition, LLC-PK1 cells derived from pig kidney and MDCK cells from dog kidney (proximal and distal tubular epithelium, respectively) were included for inter-species comparison. f_u,cell_ was first determined for 19 structurally diverse drugs (Fig. 1b, Table S1 and Fig. S2). The drug selection was based on a principal component analysis (PCA) using 1146 drugs and 334 predicted ADMET-related molecular properties (Fig. 1b), to assure that a wide range of physico-chemical properties were covered (MW: 194 to 629, PSA: 28 to 146 Å, logD_7.4_, −0.7 to 5.0; Table S1) (6).

The f_u,cell_ values, determined using membrane equilibrium dialysis, spanned four orders of magnitude and followed a similar pattern for all cell types, but with an average 9.3-fold difference between the maximum and minimum values for the different cell types (Fig. 1c). In general, the highest f_u,cell_-values were observed in the HL60 and K562 cell lines and the lowest f_u,cell_-values in HH. For all cell types, f_u,cell_ was related to the lipophilicity of the compounds, and the geometric mean values of f_u,cell_ across all cell types were negatively correlated to the logD values(R^2^ = 0.65; Fig. 1d) (5). No correlation was observed between f_u,cell_ and f_u,plasma_ (15) (Fig. 1e). In the three kidney-derived cell lines (HEK293, LLC-PK1 and MDCK), the variation between cell types was, on average, lower (Fig. S3). When the two renal epithelia cell lines (LLC-PK1 and MDCK) were compared with each other, the average difference was further reduced to 1.8-fold.

### f_u,cell_ in Comparison to Total Phospholipid Content in Cells

**Fig. 2:**
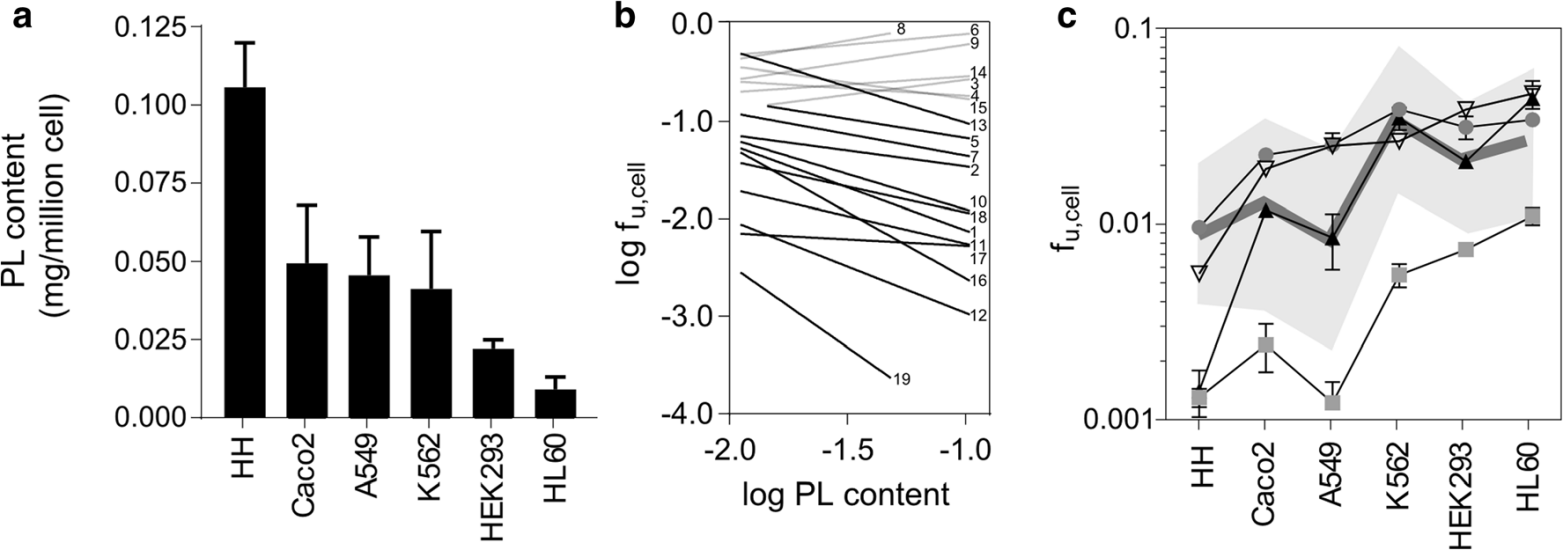
(**a**) PL content (mg/million cell) determined using an enzymatic assay for each of the human cell types. (**b**) Linear regression of f_u,cell_ and PL content. The numbers correspond to the compounds in Table S4. Compounds of the highly bound class (f_u,cell_ < 0.1, *n* = 12) are depicted in black and low binding drugs (*n* = 7) are depicted in grey. (**c**) f_u,cell_ of significantly correlated compounds in panel B (lovastatin (■), phenazopyridine (▲), atorvastatin (▽) and repaglinide (●) in all human cell types, sorted according to their PL content in panel A. The thick line represents the geometric mean of f_u,cell_ across all compounds in a given cell line, and the greyed area to the 95% confidence interval.

We previously observed a decrease in f_u,cell_ with increased PL content in the mouse fibroblast 3T3-L1 cell line (6). We hypothesized that the difference in binding between unrelated cell types could also be explained by differences in total PL content. For this purpose, we first determined total PL content per cell using an enzymatic kit and sorted the six cell types in descending order (Fig. 2a). Total PL content was then related to the median f_u,cell_ across the six cell types. Statistical significance was assessed from the linear regression of log f_u,cell_ *versus* PL content (Fig. 2b). f_u,cell_ was negatively correlated to the PL content in these cells, with statistical significance for four compounds (lovastatin, phenazopyridine, atorvastatin and repaglinide; *p* < 0.05). These compounds are highlighted in Fig. 2c. All 12 compounds with an f_u,cell_ below 0.1 (i.e. compounds that are bound more than 90%), had slopes following the same trend. The seven low binding compounds with f_u,cell_ above 0.1 (shown in grey, Fig. 2b) were not affected by PL content. Together, these results support the hypothesis that PL content is a significant contributor for unspecific cellular binding of drugs, except when the overall binding to cell membranes is low.

### f_u,cell_ in Comparison to Lipid Affinities

**Fig. 3:**
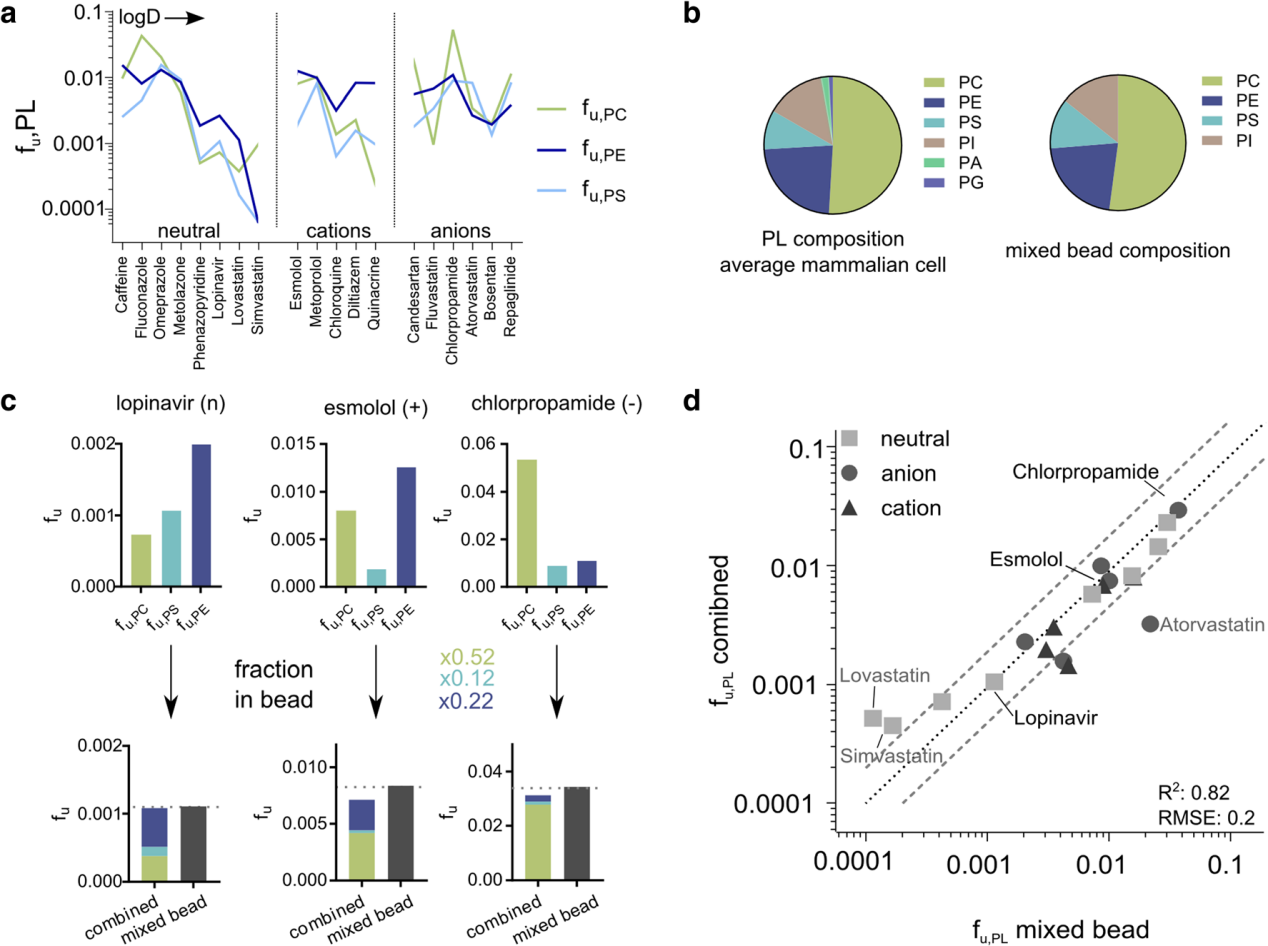
(**a**) f_u,PC_, f_u,PE_ and f_u,PS_ sorted by logD and charge class of the compounds. Numerical values are presented in Table S5. (**b**) Composition in mol% of each PL subspecies of a typical mammalian cell (17) and in the mixed beads. (**c**) Examples illustrating the additivity of drug affinities. The affinities of the individual PLs (upper panel) were multiplied with the specific fraction in the bead (0.52 for PC, 0.12 for PS and 0.22 for PE) to obtain the combined PL affinity which was compared to the one obtained in mixed beads (lower panel). Lopinavir represents a neutral compound with low f _u,PL_, esmolol a cation with intermediate f _u,PL_ and chlorpropamide an anion with high f _u,PL_. (**d**) Correlation between drug affinities measured in beads containing a combination of lipid species (f_u,PL_ mixed bead) and drug affinities calculated by combining affinities to beads containing the individual lipid species PC, PE or PS. The dotted lines indicate a 2-fold error.

Next, we investigated if the differences in f_u,cell_ could be further explained by the drug’s affinity to different PL subspecies. We therefore measured affinities of the 19 compounds to beads coated with pure PC, PE or PS membranes (Fig. 3a). These three PL subspecies were chosen because they are the major PL subspecies in a mammalian cell (Fig. 3b). We also aimed to use pure PI, but this was not possible due to the high cost of this PL. Affinities to these membranes (expressed as membrane:buffer distribution coefficients) were converted to f_u,PL_ (termed specifically f_u,PC_, f_u,PE_ and f_u,PS_ for each PL subspecies) using Eqs. 4 and 6. We observed a clear relationship of f_u,PL_ to log D for the neutral compounds (Fig. 3a). This trend was less apparent for the anionic and cationic compounds, which indicates the importance of charge interactions between the drug molecules and PL membranes. All f_u,PE_ values were confined within one order of magnitude (0.01 > f_u,PE_ > 0.001), except for the most lipophilic compound in the series (simvastatin, f_u,PE_ = 0.0001). The f_u,PC_ and f_u,PS_ values covered more evenly the range from 0.0001 to 0.05.

Next, we devised mixed beads containing all four major PL subspecies (52:22:12:14 mol% for PC:PE:PS:PI) to mimic the PL composition in a typical mammalian cell (17) (Fig. 3b). We compared f_u,PC_, f_u,PE_ and f_u,PS_ to the f_u,mixed beads_ to better understand the individual contribution of the PL subspecies to drug binding. To our satisfaction, drug affinities for PC, PE and PS were additive, i.e. the sum corresponded to the drug affinities for the mixed beads—provided that proportions of the individual PLs in the beads were considered. This is exemplified for three compounds in Fig. 3c. In this way, the drug affinities to mixed beads could be predicted from the individual drug affinities with an average error of 1.6-fold (R^2^ = 0.83; RMSE = 0.2; Fig. 3d).

**Fig. 4:**
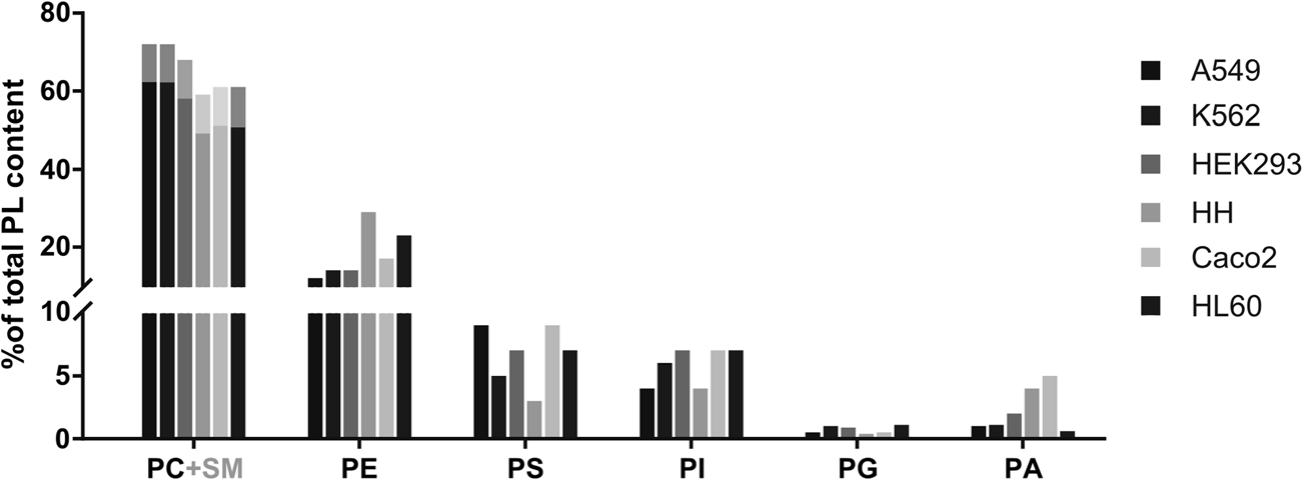
MS-based shotgun PL analysis of the six human cell types. Contents of individual PL subspecies are expressed as % of total identified PL species. PC and SM shared the same analytical fragment; an average content of 10% SM was therefore subtracted from the PC-MS signal, depicted in grey (17–19).

To better understand the contribution of individual PLs in the cell types, we determined the proportional content of each PL subspecies for each of the 8 cell types in this study, using an MS-based shotgun approach. The total intensities of each specific PL-fragment were expressed as the percentage of the total intensities of all detected PL (Fig. 4). Since PC and sphingomyelins (SM) share the same fragment, these two lipid classes were not possible to separate without an additional ion-mobility separation technique not available in our laboratory. Based on literature data of SM abundance in mammalian cells we therefore subtracted an average content of 10% SM to calculate the percentage of PCs (17–19). Overall, our results reflected average literature values for PC (49-62%), PE (12-29%), PS (3-9%), PI (4-7%), with only minor contributions from phosphatidylglycerol (PG) and phosphatidic acid (PA).

**Fig. 5:**
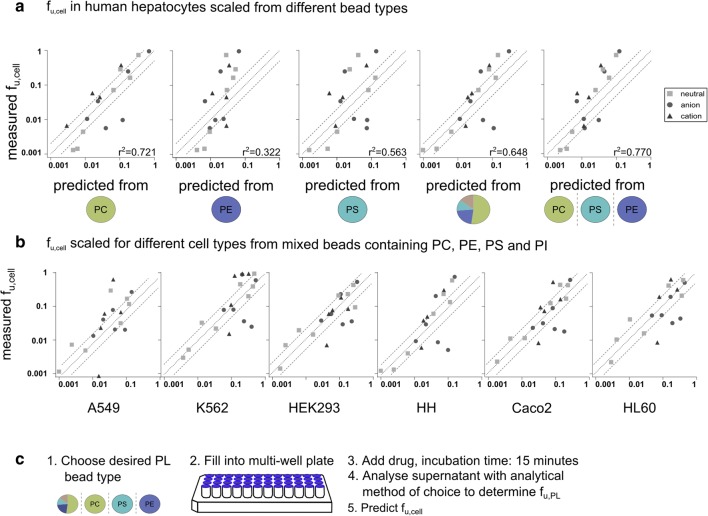
(**a**) Correlations between measured and predicted f_u,cell_ in human hepatocytes, based on affinities to the different bead types. The dotted line indicates a 2-fold error. A statistical overview (R^2^ and RMSE) is available in S7. (**b**)). Correlation of measured and predicted f_u,cell_ in the different cell types, based on the affinities to the mixed beads containing four phospholipid species (PC, PE, PS and PI). The dotted line indicates a 2-fold error. A statistical overview (R^2^ and RMSE) is available in S7. (**c**) Method overview for prediction of f_u,cell_ using PL beads.

We next used the PL composition of the cells to scale f_u,PL_ to f_u,cell_. The scaling was first performed by applying Eqs. 2 and 6 and an optimized dilution factor, D_L_, that was determined for each cell type individually (Fig. 5a). This dilution factor represents the concentration of ‘binding sites’ in a Langmuir binding isotherm model. The scaling was performed separately for f_u,PC_, f_u,PE_, f_u,PS_ and f_u,mixed bead_. Given the observed additivity of membrane affinities (Fig. 3), we also combined the affinities obtained with beads coated with single PL-subspecies using the relative content of each PL subspecies (last panel in Fig. 5a). On the basis of R^2^ and RMSE (Fig. S7), the best correlations for the different cell types were, in most cases, obtained with the mixed beads (Fig. 5b).

## Discussion

Structurally different drugs bind to cellular constituents such as lipids and proteins to varying degrees. This reduces the concentration of free drug available (f_u,cell_) for intracellular target interactions. Because measurement of cell- and tissue-specific drug binding requires invasive methodologies, the free plasma concentration has traditionally been used as a surrogate for the free tissue concentration in specific organs. However, the correlation between these two parameters is poor and therefore methods have been developed for measuring tissue-specific free concentrations (20,21). We have adapted one such methodology (22) to a format suitable for drug discovery applications (5,16).

Here, we use our small-scale, high-throughput method for determination of f_u,cell_ to systematically investigate the unbound fraction of drugs in cells (f_u,cell_) (Fig. 1a–c).

Our study indicates a significant variation of f_u,cell_ across six cell types. The difference in f_u,cell_ of 19 chemically diverse drugs was on average 9.5-fold for the human cell types (Fig. 1c) and correlates with total PL content in the cells (Fig. 2). Statistical analysis of the variance of a given compound between the six cell types derived from different organs and the three kidney-derived cell types from different species indicated significantly higher inter-organ variability than inter-species variability (*p* = 0.0001, paired t-test, S1). This is in line with previous studies that indicate low inter-species variability of drug binding in hepatocytes or brain tissue (9,23).

The correlation of f_u,cell_ across cell types with logD (Fig. 1d) was also in-line with previous findings (5). Indeed, current in silico models for prediction of f_u,cell_ rely on this parameter (24). However, the correlation with lipophilicity did not explain the spread of maximal and minimal f_u,cell_ values for a given compound in the different cell types (Fig. 1d). Instead, this spread was explained by the PL concentration. Thus, cells with the highest PL content (HH) had the lowest f_u,cell_ values and the cells with the lowest PL (HL60) contents had high f_u,cell_ values (Fig. 2).

In this study, f_u,cell_ was determined using the binding method in cell homogenates at a cell concentration of 10 million cells/ml. At this concentration, accurate determination of f_u,cell_ (accepting an error of 15%) is possible for f_u,cell_ values below 0.1. (16,23). For f_u,cell_ values above 0.1, gradually larger errors are obtained. If desired, this error can be reduced by increasing the cell concentration (23). This is because the experimental error deceases with decreasing dilution of the cells (represented by the dilution factor D_P_ in Eq. 2). In our set-up, D_P_ is determined from protein measurements in each experiment, to account for experimental variation in cell number and differences in cell volume between different cell types. After normalization for cell number and volume, we still observe differences in binding among the various cell types, throughout the whole range of f_u,cell_-values (0.0001 to 1, Fig. 1c). Therefore, we conclude that the differences of f_u,cell_ between cell types is not explainable merely by dilution effects, as recently suggested (23).

A second dilution factor, D_L,_ that accounts for differences in binding capacity among the different cell types, was used to scale f_u,cell_ from the f_u,PL_ that was determined from the beads with immobilized pure PLs (Eq. 6). Cell-type specific D_L_ values were optimized for each PL bead type by minimizing the sum of the squared prediction errors. This approach has been used previously to predict f_u,brain_ from binding to cellular homogenates (9). The optimized D_L_ values from the mixed PL beads were in good agreement with the D_L_ values that took the proportional content of PL subspecies into account. This reflects the additive properties of the bead affinities (Table S8). In line with our hypothesis that PLs are the major binding site of drugs, we observed that HH, which had the highest PL content, had the lowest D_L_.

For most cell types, the best correlations with a single PL species were obtained with PC (Fig. S7). This was not surprising, given that PC is by far the most abundant PL species in cellular membranes (>50%, Fig. 4). An exception was the Caco-2 cell line, for which the best correlation was with f_u,PS_ (Fig. S7). Interestingly, this cell line had one of the highest PS contents (~9%). Previous studies on drug interactions with pure PLs indicate that binding affinities of amine-containing basic compounds can be more than hundred fold higher for PS than for PC (25). Thus, despite its lower abundance, the PS content may influence overall binding more than might be expected on the basis of its membrane concentration. However, the results in the Caaco-2 cells were not in agreement with those in A549 cells, that also had a high PS content. Factors not covered in this investigation could contribute to these differences. These include contributions from other PL-derived lipid species or differences in subcellular or even local membrane distribution of the different PL species. Cellular components not yet considered could also contribute to the discrepancy, e.g. glycogen depots that increase with time in long term cultures of Caco-2 cells. The affinity of the different drugs to PE was confined within a fairly narrow range (1 log unit, Fig. 3a) except for of the most lipophilic drug of the data set (simvastatin). Thus, the discriminative power of pure PE was low, and the values scaled from f_u,PE_ gave the poorest correlations with f_u,cell_ (Fig. S7).

The best correlations between binding in cells and binding to PL-coated beads were obtained when contributions from several PL subspecies were combined (Fig. 5b). However, in this system, the f_u,cell_ of three compounds (atorvastatin, repaglinide and quinacrine) was consistently over-predicted. This could not be explained by common physicochemical properties or PL affinity. Further studies are required to explain these results.

The PL profiling did not reveal any outlier in PL composition between the cell types. Similar standard culture conditions were used throughout, and it is widely recognized that membrane compositions of cells are affected by culture conditions (19,26). Therefore, PL content is likely to differ to a larger extent in primary cells that require specialized culture media developed to better reflect their tissue-specific environments. Variation in lipid composition in cells is also associated with diseased states, (27) which could lead to altered drug distribution and f_u,cell_. Given the complexity of the cellular lipidome (>1000 different lipid molecules in the plasma membrane alone (28)), more detailed studies are required to elucidate these issues. In this context, it is important to note that cellular proteins contribute to non-specific cellular drug binding to a much smaller extent than PLs; even in hepatocytes, where albumin is synthesized (6,29).

In summary, the cell-free approach for determination of f_u,cell_ introduced in this contribution has several experimental advantages. These include a reduced equilibration time compared to the cellular assay (15 *vs*. 240 min) and the possibility to reduce the dilution factor by increasing the number of beads. A lower dilution factor will most likely reduce the experimental errors, as observed for cell homogenates (23). Further, once the correlation to the cell type of interest has been established (in terms of D_L_), the same batch of PL beads can be used, reducing experimental variability. The PL bead assay can be performed at different levels of sophistication. In its simplest form, PC-beads—representing the most abundant PL species (>50%) in cells—can be used to obtain an approximation of f_u,cell_. In a more advanced variation, beads can be constructed that are composed of the most abundant PLs in proportions representing an average or a specific human cell, as exemplified by the mixed PL beads in this study. However, this approach will require custom-made beads for each cell type. As a more flexible alternative, a series of PL-beads representing each of the most common PLs can be constructed and then combined in proportions representing different cell types. Further optimization will be possible, e.g. by incorporation of cholesterol as a major membrane component, and by deconvolution of subcellular PL distribution. Our results show that f_u,cell_ can be predicted by distribution into phospholipid beads. This raises the question if a cell free methodology also can be devised for determination of Kp. However, this will probably be more difficult since Kp is influenced by (sometimes unknown) cell dependent mechanisms such as active uptake and efflux transport and metabolic processes that will be difficult to mimic in a cell-free system.

## Conclusion

In conclusion, our results indicate that, independently of cell type, the cellular PL content determines to a large extent the free cellular fraction of drugs available for interaction with intracellular targets. The PL content and composition differ between cell types and correlate to f_u,cell_. We also found that f_u,cell_ determined in cell homogenates can be predicted from drug affinities to PL membranes when appropriate dilution factors are applied. We therefore devised PL-covered beads that better represent the cellular contents than beads containing merely PC. These beads are a promising approach for a high-throughput and cell-free prediction of f_u,cell_.

### ACKNOWLEDGMENTS AND DISCLOSURES

Open access funding provided by Uppsala University. We thank Sovicell GmbH for customizing and providing the phospholipid beads used in this study. We thank Simulations Plus for access to the ADMET Predictor software and ChemAxon for access to the JChem Suite. Funding: Swedish Research Council, grants no. 2822 and 2017-01951 (Per Artursson); ARIADME, a European FP7 ITN Community’s Seventh Framework Program, grant no. 60751 (Andrea Treyer); The Swedish Fund for Research without Animal Experiments, Magnus Bergvall Foundation, Åke Wiberg Foundation (Pär Matsson).

## Electronic supplementary material

ESM 1 (PDF 1523 kb)

## ABBREVIATIONS

A549: Adenocarcinomic human alveolar basalepithelial cells
Caco-2: Human epithelial colorectal adenocarcinoma cells
DMEM: Dulbecco’s Modified Eagle’s Medium
FBS: Fetal bovine serum
f_u,cell_: Intracellular fraction of unbound compound
HEK293: Human embryonic kidney 293 cells
HH: Human hepatocytes
HL-60: Human leukemia cell line
K562: Myelogenous leukemia cells derived from bone marrow
LLC-PK1: Lilly Laboratories Cell-Porcine Kidney 1
logD_7.4_: Octanol-buffer distribution coefficient at pH 7.4
MDCK: Madin-Darby canine kidney cells
MW: Molecular weight
PA: Phosphatidic acid
PC: Phosphatidylcholine
PCA: Principal component analysis
PE: Phosphatidylethanolamine
PEST: Penicillin-streptomycin
PG: Phosphatidylglycerol
PI: Phosphatidylinositol
PL: Phospholipid
PS: Phosphatidylserine
PSA: Polar surface area
RMSE: Root mean square error
