# Supplementary material for: A Cell-Free Approach Based on Phospholipid Characterization for Determination of the Cell Specific Unbound Drug Fraction (fu,cell)
Source: Pharm Res. 2019 Nov 7;36(12):178. doi: 10.1007/s11095-019-2717-1 (PMC6838048; doi:10.1007/s11095-019-2717-1)
Supplement: Supplementary file 1 — (PDF 1523 kb) [file 11095_2019_2717_MOESM1_ESM.pdf]

Supplementary material

A cell-free approach based on phospholipid characterization for determination of the cell specific unbound drug fraction ($f_{u,cell}$)

Treyer, Andrea[1]; Walday, Sandra[1]; Boriss, Hinnerk[2]; Matsson, Pär[1]; Artursson, Per[1,3,4]*

[1]Department of Pharmacy, Uppsala University, Uppsala, Sweden

[2]HBO consult GmbH, Leipzig 04155, Germany

[3]Science for Life Laboratory Drug Discovery and Development platform (SciLifelab DDD-P), Uppsala Sweden

[4]Uppsala University Drug Optimization and Pharmaceutical Profiling Platform (UDOPP), Uppsala University, Uppsala, Sweden

Content:

S 1 Overview of compound properties

S 2 Chemical structures

S 3 $f_{u,cell}$ in HEK293, MDCK and LLC-PK1

S 4 Overview of $f_{u,cell}$ values of 19 compounds in 8 cell types

S 5 Overview of bead affinities of 10 compounds in 4 bead types

S 6 Standard deviation of $f_{u,cell}$ in human vs kidney cells

S 7 Correlations: Scaling from PC, PS, PE, mixed beads, and lipidomic-informed proportions

S 8 Dilution factors D_L used for scaling $f_{u,PL}$ to $f_{u,hom,pred}$

S 9 LC-MS parameters

S 1 Overview of compound properties

Compound	$logD_{7.4}$	PSA	MW	charge at pH 7.4	Smiles
Atorvastatin	2.2	112	558.6	-	CC(C)C1=C(C(=O)NC2=CC=CC=C2)C(=C(N1CC[C@@H](O)C[C@@H](O)CC(O)=O)C1=CC=C(F)C=C1)C1=CC=CC=C1
Bosentan	2.79	146	551.6	-	COC1=CC=CC=C1OC1=C(NS(=O)(=O)C2=CC=C(C=C2)C(C)(C)C)N=C(N=C1OCCO)C1=NC=CC=N1
Candesartan	-0.65	119	440.5	-	CCOC1=NC2=CC=CC(C(O)=O)=C2N1CC1=CC=C(C=C1)C1=CC=CC=C1C1=NNN=N1
Chlorpropamide	1.49	75.3	276.7	-	CCCNC(=O)NS(=O)(=O)C1=CC=C(Cl)C=C1
Fluvastatin	1.06	82.7	411.5	-	CC(C)N1C(\C=C\[C@H](O)C[C@H](O)CC(O)=O)=C(C2=CC=CC=C12)C1=CC=C(F)C=C1
Repaglinide	3.12	78.9	452.6	-	CCOC1=CC(CC(=O)N[C@@H](CC(C)C)C2=CC=CC=C2N2CCCCC2)=CC=C1C(O)=O
Chloroquine	2.42	28.2	319.9	+	CCN(CC)CCCC(C)NC1=C2C=CC(Cl)=CC2=NC=C1
Diltiazem	2.67	59.1	414.5	+	COC1=CC=C(C=C1)[C@@H]1SC2=CC=CC=C2N(CCN(C)C)C(=O)[C@@H]1OC(C)=O
Esmolol	-0.28	67.8	295.4	+	COC(=O)CCC1=CC=C(OCC(O)CNC(C)C)C=C1
Metoprolol	-0.13	50.7	267.4	+	COCCC1=CC=C(OCC(O)CNC(C)C)C=C1
Quinacrine	3.16	37.4	400.0	+	CCN(CC)CCCC(C)NC1=C2C=C(OC)C=CC2=NC2=CC(Cl)=CC=C12
Caffeine	-0.15	61.8	194.2	n	CN1C=NC2=C1C(=O)N(C)C(=O)N2C
Fluconazole	0.82	81.7	306.3	n	OC(CN1C=NC=N1)(CN1C=NC=N1)C1=CC=C(F)C=C1F
Lopinavir	3.53	120	628.8	n	CC(C)[C@H](N1CCCNC1=O)C(=O)N[C@H](C[C@H](O)[C@H](CC1=CC=CC=C1)NC(=O)COC1=C(C)C=CC=C1C)CC1=CC=CC=C1
Lovastatin	4.58	72.8	404.6	n	CC[C@H](C)C(=O)O[C@H]1C[C@@H](C)C=C2C=C[C@H](C)[C@H](CC[C@@H]3C[C@@H](O)CC(=O)O3)[C@@H]12
Metolazone	2.25	92.5	365.8	n	CC1NC2=CC(Cl)=C(C=C2C(=O)N1C1=CC=CC=C1C)S(N)(=O)=O
Omeprazole	1.9	77.1	345.4	n	COC1=CC=C2N=C(NC2=C1)S(=O)CC1=NC=C(C)C(OC)=C1C
Phenazopyridine	2.6	89.7	213.2	n	NC1=CC=C(\N=N\C2=CC=CC=C2)C(N)=N1
Simvastatin	4.98	72.8	418.6	n	CCC(C)(C)C(=O)O[C@H]1C[C@@H](C)C=C2C=C[C@H](C)[C@H](CC[C@@H]3C[C@@H](O)CC(=O)O3)[C@@H]12

S 2 Chemical structures

Atorvastatin

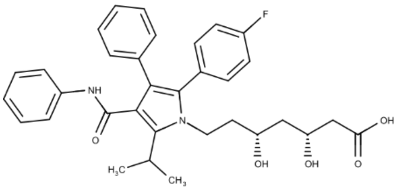

Bosentan

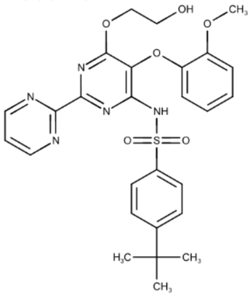

Caffeine

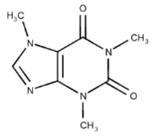

Candesartan

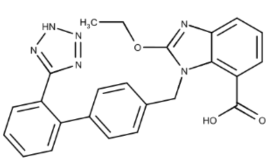

Chloroquine

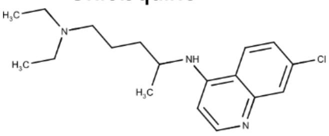

Chlorpropamide

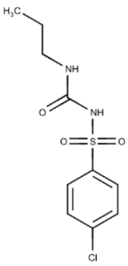

Diltiazem

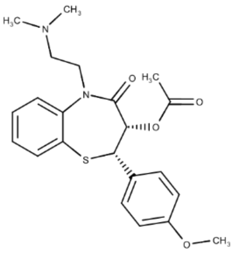

Esmolol

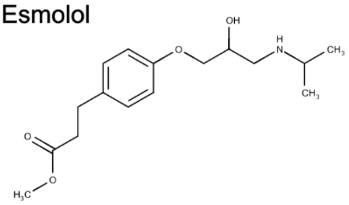

Fluconazole

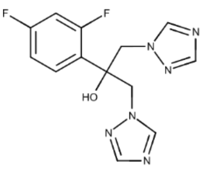

Fluvastatin

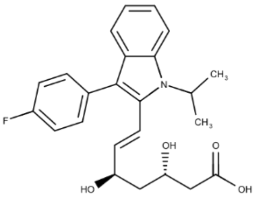

Lopinavir

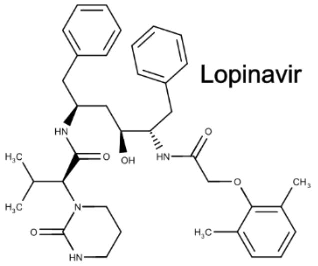

Lovastatin

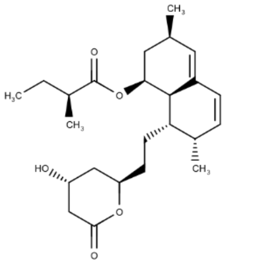

Metolazone

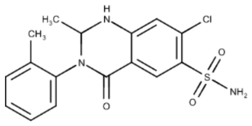

Metoprolol

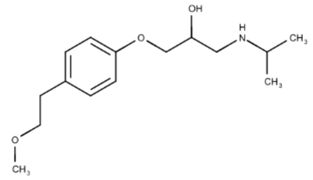

Omeprazole

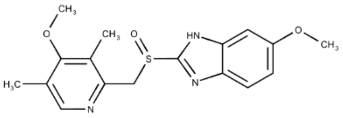

Phenazopyridine

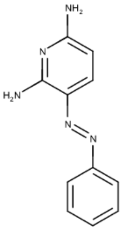

Quinacrine

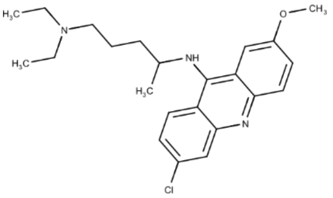

Repaglinide

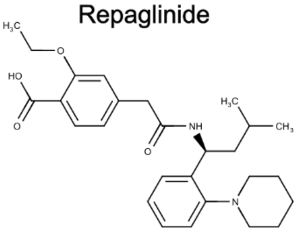

Simvastatin

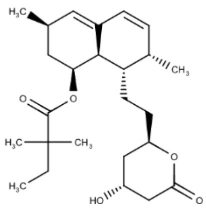

S 3 $f_{u,cell}$ in HEK293, MDCK and LLC-PK1

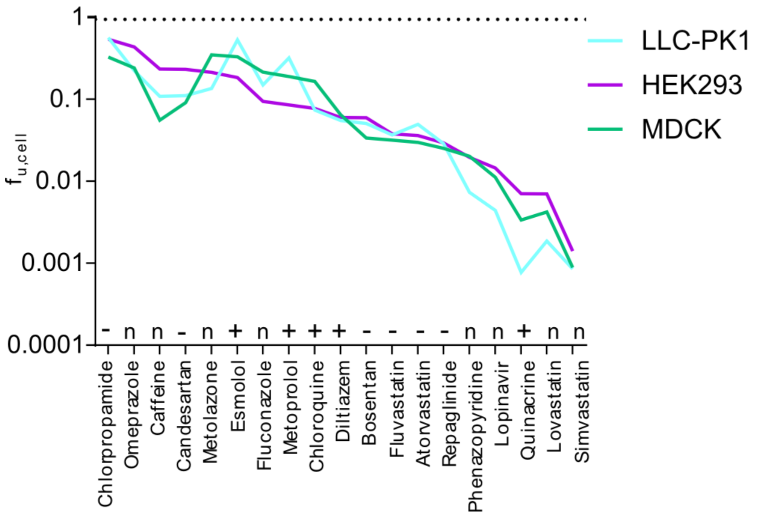

In the three kidney-derived cell lines (HEK293, LLC-PK1 and MDCK), the variation between cell types was, on average, 2.7-fold. When the two renal epithelia cell lines (LLC-PK1 and MDCK) were compared with each other, the average difference was further reduced to 1.8-fold.

S 4 Overview of $f_{u,cell}$ values of 19 compounds in 8 cell types

	$f_{u,HEK293}$		$f_{u,HL60}$		$f_{u,Caco-2}$		$f_{u,LLCPK1}$		$f_{u,A549}$		$f_{u,K562}$		$f_{u,MDCK}$		$f_{u,HH}$	
	Geomean	SD	Geomean	SD	Geomean	SD	Geomean	SD	Geomean	SD	Geomean	SD	Geomean	SD	Geomean	SD
Atorvastatin	0.0384	0.0035	0.0463	0.0074	0.0191	0.0009	0.0525	0.0103	0.0250	0.0040	0.0265	0.0008	0.0318	0.0050	0.0056	0.0003
Bosentan	0.0634	0.0076	0.0579	0.0075	0.0335	0.0004	0.0538	0.0108	0.0519	0.0141	0.0856	0.0050	0.0360	0.0064	0.0337	0.0030
Caffeine	0.2476	0.2335	ND		0.4665	0.4050	0.1151	0.0480	0.0398	0.0042	0.2142	0.3334	0.0593	0.0262	0.2476	0.2845
Candesartan	0.2465	0.1052	0.2048	0.1875	0.0945	0.0273	0.1176	0.0626	0.1033	0.0219	0.9476	0.0721	0.0968	0.0469	0.2466	0.1070
Chloroquine	0.0824	0.0310	ND		0.0777	0.0040	0.0783	0.0528	0.0793	0.0006	0.3559	0.4189	0.1756	0.1834	0.0824	0.0581
Chlorpropamide	0.5711	0.9101	0.5400	0.1275	0.6543	0.6024	0.6004	0.2549	0.3764	0.4373	0.6381	0.4274	0.3486	0.3059	0.9438	0.7714
Diltiazem	0.0637	0.0112	0.2064	0.2234	0.1004	0.0041	0.0588	0.0081	0.0287	0.0216	0.1189	0.0404	0.0690	0.0169	0.0445	0.0065
Esmolol	0.1961	0.1059	0.6763	0.0828	0.5662	0.5340	0.5661	0.2266	0.9223	0.7367	1	0	0.3499	0.5967	ND	
Fluconazole	0.1001	0.0210	0.6464	0.1392	0.4556	0.4471	0.1585	0.1422	0.1590	0.1355	1	0	0.2286	0.2147	0.7371	0.4592
Fluvastatin	0.0403	0.0043	0.0569	0.0112	0.0245	0.0030	0.0389	0.0024	0.0161	0.0036	0.0838	0.0030	0.0338	0.0074	0.0104	0.0034
Lopinavir	0.0155	0.0019	0.0168	0.0017	0.0122	0.0006	0.0047	0.0002	0.0056	0.0008	0.0234	0.0008	0.0119	0.0010	0.0044	0.0006
Lovastatin	0.0074	0.0004	0.0110	0.0011	0.0024	0.0007	0.0020	0.0001	0.0012	0.0003	0.0055	0.0007	0.0045	0.0003	0.0013	0.0001
Metolazone	0.2258	0.1068	0.4448	0.5304	0.1327	0.0582	0.1445	0.0838	0.4164	0.6967	0.4846	0.3683	0.3703	0.3015	0.0709	0.0155
Metoprolol	0.0906	0.0061	0.3391	0.2009	0.1740	0.0876	0.3402	0.3291	0.0881	0.0427	1	0	0.2019	0.2861	0.3739	0.1876
Omeprazole	0.4629	0.3475	0.2228	0.0405	0.1811	0.0436	0.2377	0.1772	0.2320	0.3079	0.4187	0.4795	0.2554	0.1810	0.1613	0.0427
Phenazopyridine	0.0208	0.0009	0.0438	0.0051	0.0118	0.0004	0.0078	0.0002	0.0085	0.0027	0.0348	0.0046	0.0215	0.0024	0.0014	0.0004
Quinacrine	0.0075	0.0003	0.0090	0.0004	0.0087	0.0003	0.0008	0.0000	0.0009	0.0002	0.0164	0.0013	0.0036	0.0002	0.0066	0.0002
Repaglinide	0.0312	0.0042	0.0341	0.0007	0.0225	0.0007	0.0305	0.0044	0.0254	0.0004	0.0385	0.0005	0.0269	0.0007	0.0097	0.0006
Simvastatin	0.0015	0.0001	0.0023	0.0001	0.0002	0.00003	0.0009	0.0001	0.0002	0.00002	0.0032	0.0009	0.0009	0.0001	ND	

S 5 Overview of bead affinities of 10 compounds in 4 bead types

	$f_{u,PC}$		$f_{u,PS}$		$f_{u,PE}$		$f_{u,mixed\ bead}$	
	Geomean	SD	Geomean	SD	Geomean	SD	Geomean	SD
Atorvastatin	0.0034	0.0015	0.0083	0.0087	0.0027	0.0013	0.0203	0.0177
Bosentan	0.0020	0.0006	0.0014	0.0004	0.0019	0.0010	0.0040	0.0008
Caffeine	0.0096	0.0053	0.0025	0.0018	0.0154	0.0100	0.0145	0.0130
Candesartan	0.0174	0.0070	0.0018	0.0012	0.0055	0.0047	0.0081	0.0042
Chloroquine	0.0014	0.0011	0.0006	0.0002	0.0032	0.0030	0.0044	0.0024
Chlorpropamide	0.0534	0.0780	0.0089	0.0025	0.0109	0.2228	0.0343	0.0462
Diltiazem	0.0023	0.0009	0.0016	0.0003	0.0083	0.0091	0.0034	0.0008
Esmolol	0.0080	0.0032	0.0019	0.0007	0.0126	0.0099	0.0084	0.0069
Fluconazole	0.0428	0.0038	0.0045	0.0019	0.0081	0.0065	0.0280	0.0040
Fluvastatin	0.0010	0.0002	0.0034	0.0019	0.0067	0.0065	0.0020	0.1416
Lopinavir	0.0007	0.0001	0.0011	0.0005	0.0026	0.0016	0.0011	0.0005
Lovastatin	0.0004	0.0002	0.0002	0.0019	0.0011	0.0062	0.0002	0.0007
Metolazone	0.0059	0.0021	0.0093	0.0048	0.0085	0.0042	0.0069	0.0421
Metoprolol	0.0101	0.0065	0.0081	0.0026	0.0099	0.0037	0.0148	0.0067
Omeprazole	0.0203	0.0040	0.0154	0.0081	0.0131	0.0094	0.0237	0.0063
Phenazopyridine	0.0005	0.0001	0.0006	0.0025	0.0019	0.0007	0.0004	0.0001
Quinacrine	0.0002	0.0007	0.0010	0.0005	0.0082	0.0068	0.0029	0.0015
Repaglinide	0.0114	0.0221	0.0084	0.0082	0.0039	0.0020	0.0095	0.0065
Simvastatin	0.0010	0.0002	0.0001	0.00004	0.0001	0.00001	0.0001	0.00002

S 6 Standard deviation of $f_{u,cell}$ in human vs kidney cells

SD has been calculated for each compound with all $f_{u,cell}$ values in all cell types to assess the variability of $f_{u,cell}$ between different cells. Human cells include HEK293, A549, K562, HL-60, Caco-2 and HH. Kidney cells include HEK293, LLC-PK1 and MDCK cells.

	SD human	SD kidney
Chlorpropamide	0.130	0.130
Fluconazole	0.399	0.180
Metoprolol	0.408	0.289
Caffeine	0.406	0.311
Candesartan	0.361	0.214
Omeprazole	0.190	0.159
Metolazone	0.337	0.205
Chloroquine	0.312	0.196
Diltiazem	0.310	0.035
Bosentan	0.160	0.127
Fluvastatin	0.342	0.040
Repaglinide	0.217	0.035
Quinacrine	0.432	0.488
Atorvastatin	0.325	0.110
Lopinavir	0.285	0.273
Phenazopyridine	0.541	0.250
Lovastatin	0.402	0.289
Esmolol	0.284	0.230
Simvastatin	0.598	0.121

S 7 Correlations: Scaling from PC, PS, PE, mixed beads, and lipidomic-informed proportions

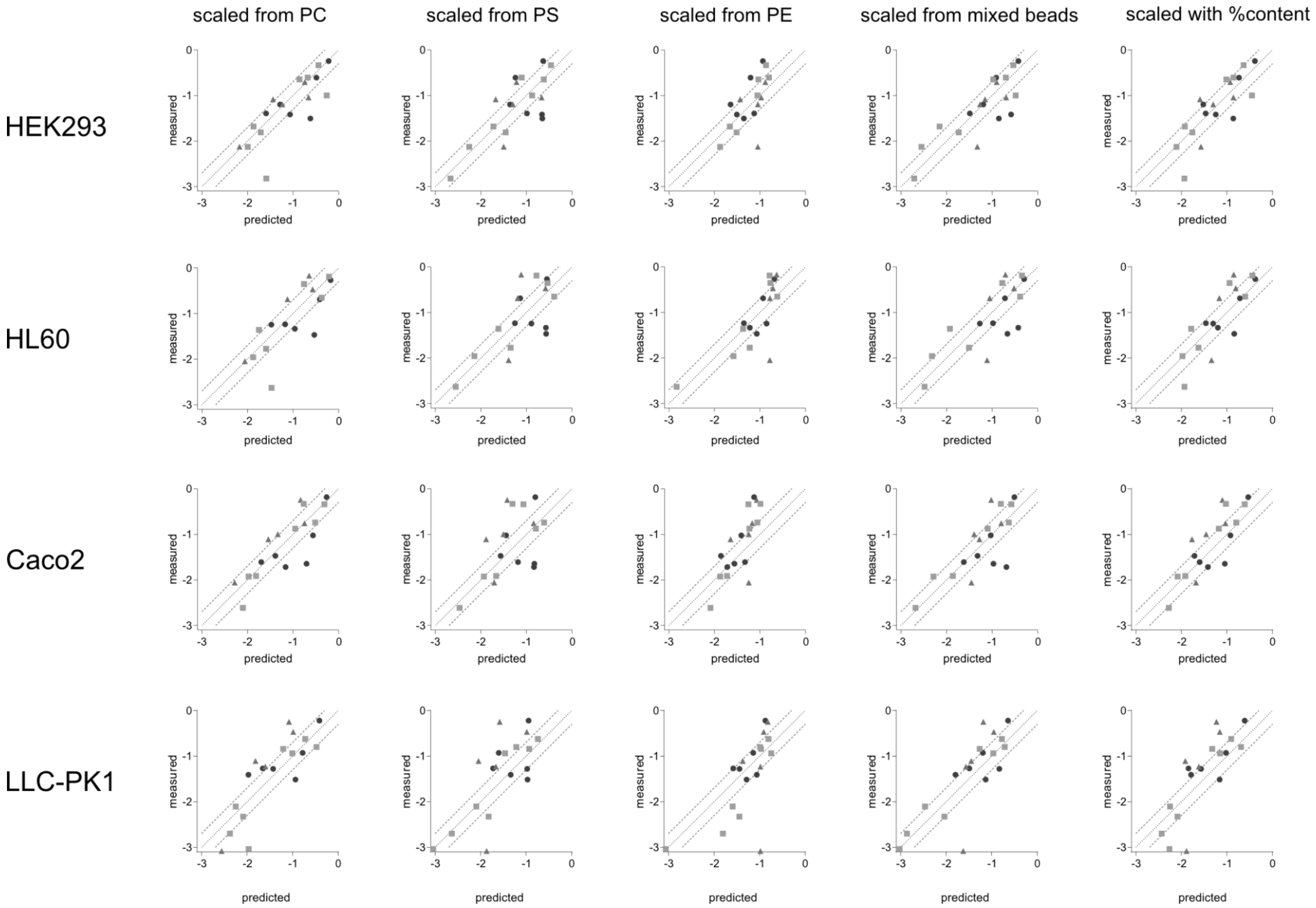

Correlations: Scaling from PC, PS, PE, mixed beads, and lipidomic-informed proportions (continued)

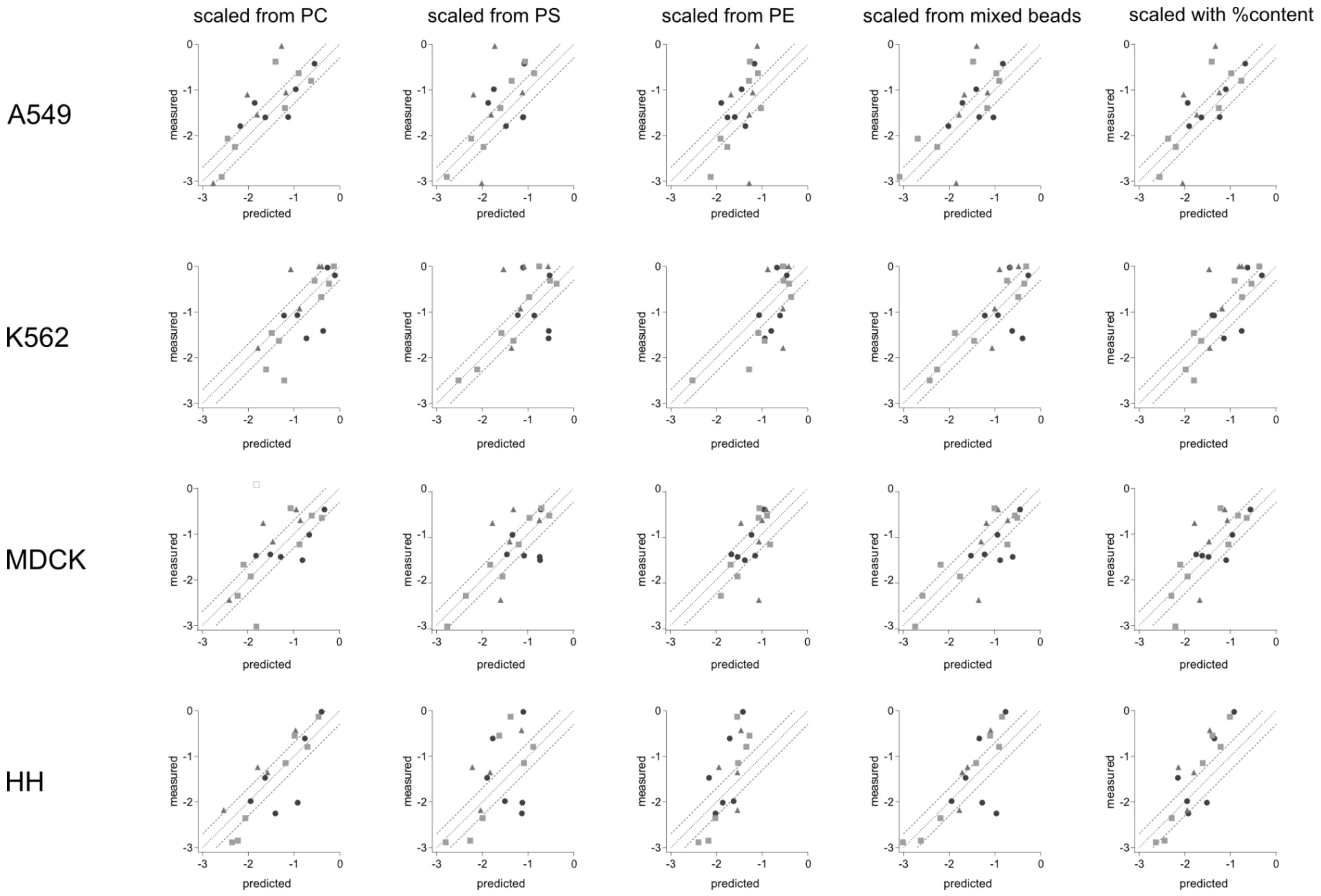

Correlations: Scaling from PC, PS, PE, mixed beads, and lipidomic-informed proportions (continued)

Overview of R2 and RMSE values of correlations presented in the plots above.

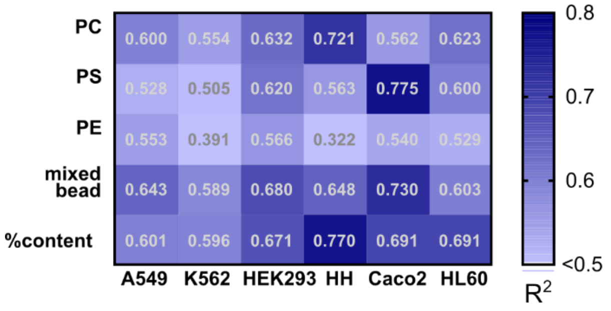

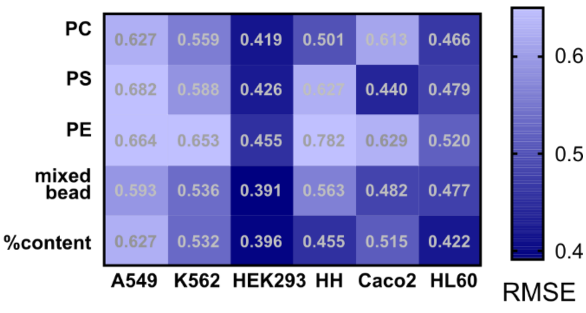

S 8 Dilution factors D_L used for scaling $f_{u,PL}$ to $f_{u,hom,pred}$

For better appreciation, the numbers are presented as $1/D_L$.

	PC	PS	PE	Mixed beads	%content
HEK293	924.1	1134.7	401.8	577.6	683.2
HL60	1124.9	1362.0	745.6	907.7	714.3
Caco2	589.9	566.8	200.3	346.9	369.3
LLC-PK1	498.4	627.7	621.5	369.0	401.5
A549	240.0	344.2	227.3	169.8	249.9
K562	2062.8	1435.5	1479.6	1006.8	808.8
MDCK	617.1	1038.5	438.9	619.0	451.8
HH	200.7	160.7	60.4	98.4	75.8

S 9 LC-MS parameters

Compound quantification was performed using UPLC-MS/MS. The system consisted of a Waters Xevo TQ MS with electrospray coupled to a Waters Acquity UPLC. Compounds were chromatically separated on a Water BEH C18 column (2.1 x 50 mm; 1.7 µm) with a 2 min gradient elution at a flow rate of 0.5 ml/min.

Compound	Retention Time (min)	Parent (m/z)	Cone Voltage	Daughter (m/z)	Collision Energy	Ionization Mode
Atorvastatin	1.47	559.2	30	202.1	32	ESI+
Bosentan	1.44	552.1	42	202.0	32	ESI+
Caffeine	0.95	195	32	138.0	18	ESI+
Candesartan	1.3	441.4	18	263.2	12	ESI+
Chloroquine	0.95	320.1	30	247.0	20	ESI+
Chlorpropamide	1.33	275.1	26	189.7	18	ESI-
Diltiazem	1.19	415.2	20	178.0	26	ESI+
Esmolol	1.16	295.9	34	218.8	20	ESI+
Fluconazole	1.13	307.1	24	238.1	16	ESI+
Fluvastatin	1.47	410.4	26	348.3	16	ESI-
Lopinavir	1.52	629.5	22	155.1	46	ESI+
Lovastatin	1.63	405.4	18	199.2	12	ESI+
Metolazone	1.24	365.9	22	258.6	20	ESI+
Metoprolol	1.38	268.2	26	116.0	18	ESI+
Omeprazole	1.17	346.2	16	198.1	12	ESI+
Phenazopyridine	1.15	213.8	28	121.8	20	ESI+
Quinacrine	1.06	400.2	24	142.1	22	ESI+
Repaglinide	1.4	453.3	32	230.2	28	ESI+
Simvastatin	1.57	435.2	34	319.2	16	ESI-
Warfarin (internal standard)	1.42	307.2	40	161.0	22	ESI+
